# Protolysis and Complex Formation of Organophosphorus Compounds—Characterization by NMR-Controlled Titrations

**DOI:** 10.3390/molecules24183238

**Published:** 2019-09-05

**Authors:** Gerhard Hägele

**Affiliations:** Institute of Inorganic Chemistry and Structural Chemistry, Heinrich-Heine-University Düsseldorf, Universitätsstraße 1, D-40225 Düsseldorf, Germany; haegele@uni-duesseldorf.de

**Keywords:** phosphonic acids, aminophosphonic acids, phosphonocarboxylic acids, NMR-controlled titration, dissociation constants, stability constants, dynamic and specific NMR parameters

## Abstract

Phosphonic acids, aminophosphonic acids, and phosphonocarboxylic acids are characterized by an advanced hyphenated technique, combining potentiometric titration with NMR spectroscopy. Automated measurements involving ^13^C, ^19^F and ^31^P nuclei lead to “pseudo 2D NMR” spectra, where chemical shifts or coupling constants are correlated with analytical parameters. Dissociation constants, stability constants, dynamic and specific chemical shifts are determined. Macroscopic and microscopic dissociation equilibria are discussed.

## 1. Introduction

NMR-controlled titration, also known as NMR titration, a useful tool combining NMR and analytical aspects, is based on fundamental observations in the dawn of NMR spectroscopy: “Early phosphorus NMR studies of condensed phosphates showed that raising the acidity of phosphate solutions increased the shielding of the phosphorus nucleus, causing a shift of the ^31^P resonances to higher fields by several ppm” [1]. “Later studies on the short chain condensed phosphates exhibited that the pH variations of the chemical shifts and spin-coupling constants where, when measured to sufficient precision, sensitive functions of the molecular structure and the bonding”. A first titration curve of H_3_PO_4_ shown as δ_P_ vs. pH was derived in this paper [2]. ^13^C-NMR measurements on linear aliphatic acids revealed that COOH groups in C_n_H_2n+1_COOH (n = 0 to 4) exhibit higher chemical shifts δ_C_ than COO^−^ groups of corresponding anions C_n_H_2n+1_COO^−^. A characteristic downfield shift of δ_C_ ranging from 5.1 to 4.7 ppm was observed for deprotonation by addition of tetramethylammonium hydroxide to carboxylic acids [3].

In subsequent years, those phenomena attracted the attention of numerous studies dealing with inorganic and organic phosphorus chemistry. A higher level of sophistication was achieved by combining the analytical theory of protolysis and complex formation for acids and bases with advanced NMR technologies and expanding the range of sensor nuclei to ^1^H, ^13^C, ^15^N, ^19^F, ^31^P and spin active metal nuclei.

The first NMR titration curves for phosphonoacetic acid HOOC-CH_2_-PO_3_H_2_ using ^13^C and ^31^P NMR were reported as δ_C_ vs. pH and δ_P_ vs. pH functions. For the first time, a characteristic deprotonation sequence was established: HOOC-CH_2_-PO_3_H_2_ → HOOC-CH_2_-PO_3_H^−^ → ^−^OOC-CH_2_-PO_3_H^−^ → ^−^OOC-CH_2_-PO_3_^2−^. In addition, ^1^*J*_PC_ was related to the s-electron density around the central C-atom [4].

Several decades of creative work followed those early observations. Induced by synthetic, analytical, biological, or technical aspects, interests were concentrated on several classes of organophosphorus compounds. Particular attention was drawn towards analogues of amino acids, e.g., aminophosphonic acids and strong complexing agents like NTMP (N(CH_2_PO_3_H_2_)_3_) and EDTMP ((H_2_O_3_PCH_2_)_2_NCH_2_CH_2_N(CH_2_PO_3_H_2_)_2_), which are phospha analogues of the classical complexones NTA (N(CH_2_COOH)_3_) and EDTA ((HOOCCH_2_)_2_NCH_2_CH_2_N(CH_2_COOH)_2_). Dissociation constants, stability constants for protonation and metal complex formation were studied as quoted with a few selected key papers [5,6,7,8,9,10,11,12,13,14,15,16].

Further interests concentrated on phospha analogues of carboxylic acids, e.g., phosphonocarboxylic acids and geminal bisphosphonic acids. ^31^P- and ^13^C-NMR spectra of cyclohexyl- and phenylphosphonic acid showed that chemical shifts δ_P_ and δ_C_ including coupling constants ^n^*J*_PC_ (n = 1–4) of cyclohexanephosphonic acid and benzenephosphonic acid proved to be pH-dependent [17].

A key paper in understanding the NMR titration of geminal bisphosphonate structures described three asymmetric esters of chlodronic acid (HO)_2_(O)P-CCl_2_-P(O)(OiPr)OH, (HO)_2_(O)P-CCl_2_-P(O)(OiPr)_2_, and HO(iPrO)(O)P-CCl_2_-P(O)(OiPr)_2_. Proton coupled ^31^P-NMR titration spectra revealed the coupling constants ^2^*J*_PP_ in a range between 15.6 and 17.9 Hz. This significant parameter is not accessible for the symmetric ester HO(iPrO)(O)P-CCl_2_-P(O)(OiPr)OH since this compound gives rise to a dynamic deceptively simple spectrum ranging from singlet to triplet as a result of the parent symmetric [AM_6_X]_2_ spin system [18]. ^31^P-NMR measurements at 202.5 MHz showed that the chemical shift δ_P_ of CH_3_C(OH)[P(O)(OH)_2_]_2_ (HEDP) is sensitive towards pH and the concentration of [(CH_3_)_4_N]^+^ when [(CH_3_)_4_N]Cl was used as an ion buffer [19].

The determination of high pK values (pK > 13) and low pK values (pK < 1) required specific, advanced techniques for NMR titration. Comments on measurements at high and low pH were reported [19,20]. ^1^H/^31^P NMR pH indicator series were used to eliminate the glass electrode in NMR spectroscopic pK determinations, leading to “electrodeless titrations” [21]. Comprehensive guidelines for NMR measurements for the determination of high and low pK values were given in a IUPAC Technical Report. Those sophisticated and detailed instructions should be followed for accurate analytical and NMR measurements, data evaluation and subsequent publications [22].

### 1.1. Developing Technical Setups for Automated NMR Titrations

In general, NMR titrations for various nuclei were performed in single sample techniques, which proved to be rather laborious and time consuming. For practical reasons, the number of data points were limited in those early titration curves. Hence, attempts were made to develop the technology of automated NMR titrations.

An innovative set up was constructed, which permitted the acquisition of spectra from spinning 20 mm NMR tubes, adding a solution of base under efficient mixing while monitoring the pH. This apparatus worked together with the wide-bore magnet of a Bruker CXP-300 spectrometer, yielding approximately 80 titration points within a couple of hours. This technology was successfully used to titrate H_3_PO_4_ vs. KOH and provided a smooth NMR titration curve [23].

Further progress for automated NMR titrations inside spinning 10 mm NMR tubes was described and the novel installation applied to monitor the complex formation between Tl(I)^+^ and Cl^−^ in aqueous solutions. A Bruker CXP-100 spectrometer was used, operating at 51.9 MHz for ^205^Tl [24].

Very recently, an elegant low-cost construction was developed for a gravity-driven pH adjustment inside a 5 mm NMR tube. No hardware modifications of the NMR spectrometer were requested. This technique was applied to site-specific protein pK measurements [25]. It might be useful for future studies of organophosphorus compounds.

A different route to automated NMR titrations was chosen by the Düsseldorf group. We were intrigued by the technology of 2D NMR spectra and the graphical representation of such spectra by standard spectrometer software. Hence, a hyphenated technique was envisaged, replacing the f2 axis of 2D NMR spectra by analytical parameters.

Bypass constructions were developed in several generations of increasing accuracy. A 10 mm NMR tube was attached to a special homemade insert and used with a Bruker AM 200 SY NMR spectrometer operating at 81 MHz for ^31^P NMR. This insert acted as bypass to a precision titration equipment. A series of pH dependent 1D NMR spectra were recorded and processed (using standard spectrometer software) to yield instructive “pseudo 2D NMR” spectra (e.g., in analogy to COSY spectra). Chemical shift δ_P_ data were correlated with analytical data like pH or the degree of titration τ. The technical setup and two examples are shown in [26]. Phosphaalanine was used as an example where deprotonation and complex formation with Zn^2+^ cations were observed by titrations vs. tetramethylammonium hydroxide (TMAOH) [26].

This equipment was used to characterize a series of aminomethylphosphine oxides (CH_3_)_3-n_(CH_2_NH_2_)_n_PO (n = 1–3), adding n equivalents of HCl and back-titrating vs. NaOH. Ion-specific chemical shifts δ_P_ and pK data were obtained for those aminomethylphosphine oxide bases. In addition, technical details of NMR, analytics, software concepts and programs used were described [27].

A brief overview of “^31^P NMR controlled titrations of Phosphorus-Containing Acids and Bases in Protolysis and Complex Formation” reported about the 81 MHz ^31^P{^1^H} NMR titration of phosphonoacetic acid [28]. The hardware and software concepts were shown.

Typical “pseudo 2D NMR” spectra correlating the chemical shift δ_P_ vs. the degree of titration τ were obtained for the pair of isomers 1- and 2-aminoethanephosphonic acids CH_3_-CH(NH_2_)-PO_3_H_2_ and NH_2_-CH_2_-CH_2_-PO_3_H_2_ (α-Ala-P and β-Ala-P) and for diphosphaasparaginic acid H_2_O_3_P-CH(NH_2_)-CH_2_-PO_3_H_2_ (Asp-P_2_). Macroscopic dissociation constants and ion-specific chemical shifts are reported. The *p*-aminophenylene-substituted phosphonic acid *p*-NH_2_-C_6_H_4_-PO_3_H_2_ was compared [29]. Hardware and software concepts used in NMR titration were demonstrated. A subsequent UV-controlled titration revealed the microscopic dissociation scheme of *p*-NH_2_-C_6_H_4_-PO_3_H_2_. Corresponding deprotonation patterns were discussed [30].

In practice, ^13^C-NMR titrations in single sample techniques proved to be very time consuming. Hence, it seemed advisable to use the technology described above for automated 50.29 MHz ^13^C{^1^H} or ^13^C-NMR measurements. As practical examples, the pair of isomers 1- and 2-aminoethanephosphonic acids were titrated vs. NaOH. Within this context, CH_3_-CH(NH_2_)-PO_3_H_2_ and the fluorinated analogue CF_3_-CH(NH_2_)-PO_3_H_2_ were compared using ^31^P{^1^H} and ^19^F{^1^H} NMR titrations. Replacing the CH_3_ by a CF_3_ group reduces the basicity of the NH_2_ function, which is reflected in δ_P_ vs. τ and in the δ_F_ vs. τ correlations [31].

The experimental set up described above requested individual titrations for each nucleus wanted. Hence, multinuclear studies (e.g., ^1^H and ^13^C and ^31^P) demanded high spectrometer times.

At this stage, special probe heads were developed by Bruker for another hyphenated technique combining liquid chromatography with HR NMR. In our laboratory, a Bruker LC probe head LC-TXO-NMR was successfully introduced to a DRX 500 NMR spectrometer and used for advanced NMR titrations. It became routine to run consecutively ^31^P{^1^H}, ^31^P, and ^1^H-NMR spectra for each titration step, thus saving time, gaining higher sensitivity and reducing the necessary concentrations (and amounts) of titrands. Excellent spectra resulted with a high S/N ratio and high digital resolution in the chemical shift or frequency axis.

In addition, a special ^19^F-LC probe head was available, combining ^19^F and ^1^H-NMR techniques. The high field stability of the supercon magnet allowed measurements in H_2_O solutions (without D_2_O), thus avoiding the problems with “mixed” stability and dissociation constants resulting from D_2_O/H_2_O mixed solvents.

A comprehensive report about the technical designs of NMR and analytical components, software, data evaluation, error calculations and applications was written in 2002 and incorporated into the Bruker NMR Guide collection, freely accessible for Bruker spectrometer users [32] only. This detailed review is now open for free downloads to all interested readers: (a) https://www.theresonance.com/nmr-controlled-titration-download-the-paper/, (b) https://www.bruker.com/fileadmin/user_upload/8-PDF-Docs/MagneticResonance/NMR/NMR_controlled_titration.pdf.

NMR titrations using 200 MHz and 500 MHz spectrometers were described using geminal bisphosphonic acids, e.g., HEDP and Pamidronic acid, as model systems. The TXO-HPLC probe head improved the signal-to-noise ratio of “pseudo 2D NMR” spectra and reduced the concentration of titrand required by this procedure: The following concentrations for sensor nuclei are recommended: ^1^H: 0.25–0.01 mol/L, ^13^C: 0.50–0.005 mol/L, ^19^F: 0.01–0.005 mol/L, ^31^P: 0.01–0.001 mol/L, and ^113^Cd: 0.25–0.1 mol/L. ^113^Cd NMR was used when studying protolytic and complex formation equilibria of (H_2_O_3_P-CH_2_)_2_NCH_2_CH_2_N(CH_2_PO_3_H_2_)_2_ (EDTMP).

Within this context, a special computer program MultipleNMRGraphics was developed which is able to generate four characteristic “pseudo 2D NMR” plots, e.g., δ_P_ vs. pH or δ_P_ vs. τ either as contour or as stacked plots, in black-and-white or color design [33]. Those graphics have a lower storage demand than the previously used “pseudo 2D NMR” spectra generated by the routine Bruker spectrometer software.

Some examples relevant to phosphorous chemistry and organic chemistry dealt with in [32] are listed in Table 1:

A modification of our design for automated NMR titrations shown above was adjusted to the local conditions of a Bruker 250 MHz spectrometer and applied to study the complexation of Zn^2+^, Cd^2+^ and Pb^2+^ with diazacrown ethers substituted by phosphonate groups [34].

Particular attention was drawn towards microscopic dissociation constants going back to early studies on NH_2_-CH_2_-CH_2_-NH-CH_2_-COOH. 60 MHz and 100 MHz ^1^H-NMR titrations evaluated the pH dependence of a singlet for the methylene group NH-CH_2_-COOH, while the ethylene function N-CH_2_-CH_2_-N appeared with the spectral character, changing from a deceptively simple singlet towards an AA′BB′ ([AB]_2_) system. The analytical formalism and microscopic dissociation constants were derived [35]. For deeper reading, an up-to-date and comprehensive survey on the theory and practice of proton microspeciation based on NMR-pH titrations is recommended [36].

As an example, *S*-2-amino-4-(methylphosphinoyl)butyric acid (*S*-phosphinothricine, GLUFOSINATE) HOOC-CH(NH_2_)-CH_2_-CH_2_-P(CH_3_)(O)OH was characterized by ^31^P{^1^H}- and ^1^H-NMR titrations. Microscopic dissociation and intramolecular rotational equilibria were discussed [32,37]. Within this context, a program LAOTIT was developed, which is able to simulate series of pH-dependent second-order NMR spectra. A practical example for AFGMNQ_3_X spin systems of GLUFOSINATE in a pH range from 1 to 6 was shown in [37].

**Scheme 1:**
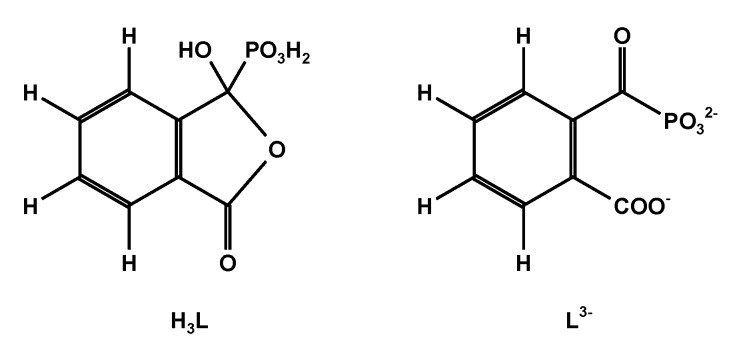
Showing 3-hydroxy-3-phosphonoisobenzofuranone H_3_L and its terminal anion L^3−^.

The ring-chain tautomerism and protolytic equilibria of an effectively three-basic 3-hydroxy-3-phosphonoisobenzofuranone was studied by ^1^H-, ^13^C{^1^H}- and ^31^P{^1^H}-NMR-controlled titrations. A complex pattern of macroscopic and microscopic deprotonation steps leading from the starting H_3_L to the final L^3−^ (Scheme 1) was discussed.

The OPIUM program enabled the simultaneous evaluation of potentiometric, ^31^P{^1^H}- and ^1^H-NMR titrations using the four individual ^1^H signals from the ABCD system [38]. Macroscopic dissociation constants, pK_1_ = 0.445 ± 0.008, pK_2_ = 5.792 ± 0.003, pK_3_ = 6.486 ± 0.002. δ_H_, δ_C_, and δ_P_ of H_3_L (0.2510 mol/L in D_2_O) and L^3−^ (0.1919 mol/L in 1 mol/L KOD), were determined. For details of the complex equilibrium system, see [39,40].

NMR-controlled titration was successfully used to analyze the mixture of diastereomers from 1-phosphonopropane-1,2,3-tricarboxylic acid, HOOC-CH_2_-CH(COOH)-CH(COOH)-PO_3_H_2_ (PPTC). The genuine product from synthesis consisted of 64% of the RS/SR and 36% of the RR/SS forms. ^31^P{^1^H}-NMR-controlled titration revealed two diastereospecific titration curves which were individually identified by additional 1D and 2D NMR studies using ^1^H, ^31^P and ^13^C nuclei. Dissociation constants and ion-specific chemical shifts δ_P_ were calculated for the pair of diastereomers [41,42,43]. It seems evident to use automated NMR titration for production control in research and industrial chemistry.

### 1.2. Some Comments on Macroscopic Protolytic Equilibria—Dissociation and Stability Constants

Organophosphorus compounds studied by potentiometric or NMR-controlled titrations may be described by two numerical indices: a, the number of acidic functions (e.g., P(O)OH, C(O)OH, etc.) and b, the number of basic functions (e.g., NH_2_, NHR, NR_2_, etc.). The minimal protonated species corresponds to the n-valent base L^−a^ having a anionic centers and b neutral base centers in (^0^N)_b_-R-(O^−^)_a_. Total protonation leads to the n-valent acid H_n_L^b+^ (n = a + b) having a neutral centers and b cationic acid centers in (^+^HN)_b_-R-(OH^0^)_a_.

Protonation equilibria of the n-valent base are described by Equation (1):

(1) $${\mathrm{iH}}^{+}+L^{-a}\overset{}{⇌}H_{i}L^{i-a}\;i\;=\;1\;\mathrm{to}\;n$$

and by brutto-stability constants following Equation (2):

(2) $$\beta_{i}=\frac{\left[H_{i}L^{i-a}\right]}{{\left[H^{+}\right]}^{i}[L^{-a}]}\;i=\;1\;\mathrm{to}\;n\;\beta_{0}=1$$

Stepwise dissociation equilibria of the n-valent acid are described by Equation (3):

(3) $$H_{n+1-i}L^{b+1-i}⇌H^{+}+H_{n-i}L^{b-i}\;i\;=\;1\;\mathrm{to}\;n$$

while corresponding dissociation constants K_i_ are given by Equation (4):

(4) $$K_{i}=\frac{\left[H^{+}][H_{n-i}L^{b-i}\right]}{\left[H_{n+1-i}L^{b+1-i}\right]}\;i\;=\;1\;\mathrm{to}\;n$$

Stoichiometric stability constants and stoichiometric dissociation constants are connected by Equations (5) and (6):

(5) $${\mathrm{pK}}_{i}=\mathrm{lg}\beta_{n+1-i}-\mathrm{lg}\beta_{n-i}\;i\;=\;1\;\mathrm{to}\;n$$

(6) $$\mathrm{lg}\beta_{i}=\sum_{j=1}^{i}pK_{n+1-j}\;i\;=\;1\;\mathrm{to}\;n$$

This paper will use stoichiometric variables (containing concentrations instead of activities) in abbreviated forms: pK_i_—macroscopic acid dissociation constant; pk_i_—microscopic acid dissociation constant; pK_w_—ion product of water. pH stands for the concentration-based pH = −lg(c_H_). Glass electrodes were calibrated by blank titration. The more complex situation of activities and activity-based parameters exceeds the scope of this paper and hence will not be discussed at this stage.

The molar fractions x_i_ of protolytic species H_i_L^i–a^ are derived from Equation (7):

(7) $$x_{i}=\frac{10^{\mathrm{lg}\beta_{i}-i\cdot pH}}{\sum_{j=0}^{n}10^{\mathrm{lg}\beta_{j}-j\cdot pH}}\;i\;=\;0\;\mathrm{to}\;n$$

Each protolytic species H_i_L^i–a^ present in the equilibrium contributes specific NMR parameters δ(H_i_L^i–a^) in an exchange reaction, which is rapid on the NMR timescale. Effectively, only one signal is observed when monitoring NMR during the course of titrations. A dynamically averaged chemical shift δ follows Equation (8):

(8) $$\delta =\sum_{i=0}^{n}x_{i}\cdot \delta_{H_{i}L^{i-a}}\;i\;=\;0\;\mathrm{to}\;n\;$$

A gradient called the deprotonation shift Δ_i_ [ppm] is given by Equation (9):

(9) $$\Delta_{i}=\delta_{H_{n-i}L^{b-i}}-\delta_{H_{n+1-i}L^{b+1-i}}\;i\;=\;1\;\mathrm{to}\;n$$

This gradient defines the change of chemical shift for each deprotonation step. Signs and magnitudes of gradients are used to elucidate the deprotonation and protonation pathways of multifunctional acids, bases and ligands as shown in examples below.

As deduced above, the dynamically averaged chemical shift δ is a function of pH. Experimentally, the pH of solutions may be varied by titration with a strong univalent base or a strong univalent acid. While the experiment directly provides the well-known titration curve pH = f(V_Titrator_), it is more convenient to calculate the inverse function V_Titrator_ = f(pH) with suitable computer programs. Within this paper, a reduced parameter τ, commonly called degree of titration, will be used to describe the status of a titration process. τ is a ratio defined by Equation (10):

(10) $$\tau =\frac{n_{Titrator}}{n_{Titrand}}$$

The sign of τ is positive if n_Titrator_ corresponds to the molar amount of a strong monovalent base (e.g., NaOH, KOH, TMAOH), but is negative for a strong monovalent acid (HCl, HNO_3_, HClO_4_). n_Titrand_ corresponds to the molar amount of a n-basic acid H_n_L.

Details of the basic principles and experimental equipment with hardware and software are described in [31,32] and in references given herein.

## 2. Results and Discussion

In the following sections, a few examples will be shown for automated NMR-controlled titrations using hardware and software concepts described above. Chemical shifts δ_C_ [ppm] quoted below were referenced vs. (CH_3_)_3_Si-CH_2_-CH_2_-SO_3_Na, while δ_P_ [ppm] was virtually referenced towards external H_3_PO_4_. Coupling constants ^n^*J*_XY_ are given in [Hz].

### 2.1. Phosphonic Acids

**Scheme 2:**
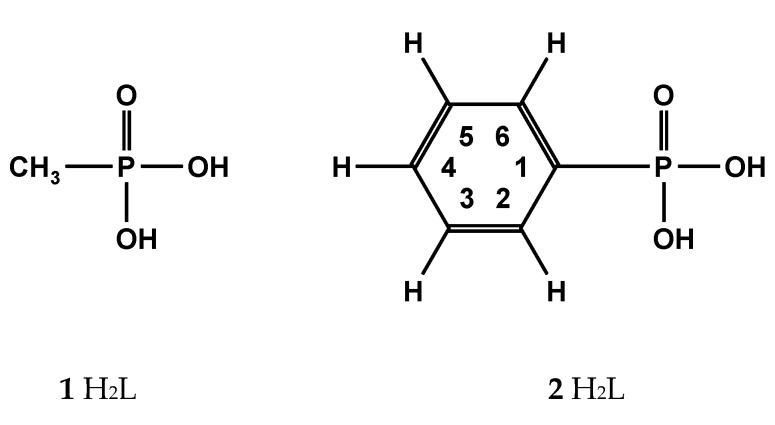
Phosphonic acids: methanephosphonic **1** and phenylphosphonic acid **2**.

Methanephosphonic acid **1** and phenylphosphonic acid **2** shown in Scheme 2 were chosen from [31,44], which will be presented below:

#### 2.1.1. Methanephosphonic Acid **1**

**Figure 1:**
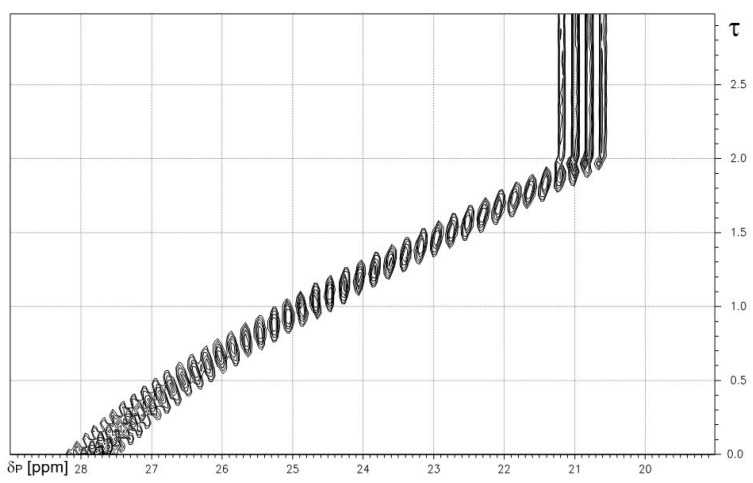
^1^H-coupled ^31^P-NMR-controlled titration of methanephosphonic acid **1** vs. NaOH. Note: quartet fine structure from X-part of AX_3_ system of P-CH_3_ fragment. *X*-axis: δ_P_ [ppm]. *Y*-axis: degree of titration τ.

The results from a proton-coupled ^31^P-NMR-controlled titration of methanephosphonic acid **2** vs. NaOH are shown as a contour plot in Figure 1. A quartet structure from the parent A_3_X spin system of the P-CH_3_ fragment is recognized. Numerical results are given in Table 2. The deprotonation of both P-OH functions induces a decrease in chemical shifts δ_P_ and a decrease in the absolute values of ^2^*J*_PH_.

pK_i_ values found are consistent with results from potentiometric titrations of CH_3_P(O)(OH)_2_ [46].

#### 2.1.2. Phenylphosphonic Acid **2**

Chemical shifts δ_P_ for protolytic species H_2_L, HL^−^, and L^2−^ of phenylphosphonic acid **2** together with dissociation constants pK_1_ and pK_2_ are listed in Table 3. The deprotonation of both P-OH groups leads to characteristic high field shifts for δ_P_ as indicated by negative gradients Δ_1_ and Δ_2_.

**Figure 2:**
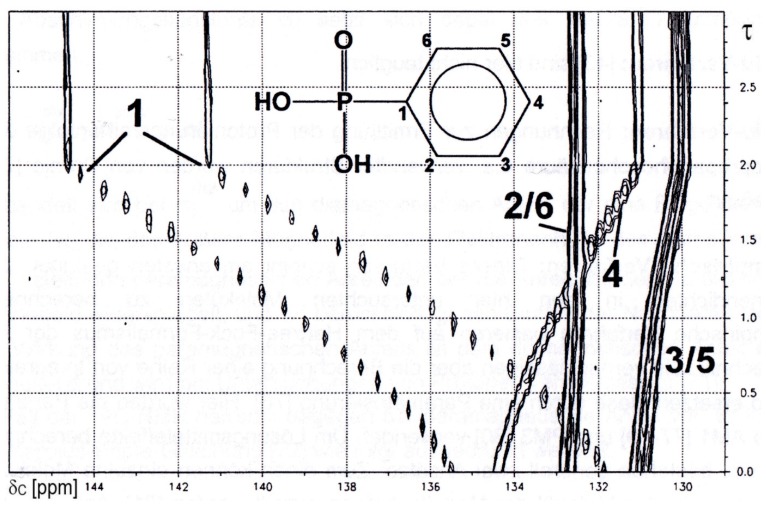
^13^C{^1^H}-NMR-controlled titration of phenylphosphonic acid **2** vs. KOH yielded a contour plot for chemical shift δ_C_ as a function of the degree of titration τ. *X*-axis: δ_C_ [ppm]. *Y*-axis: degree of titration τ.

Higher concentrations are required for ^13^C{^1^H}-NMR-controlled titrations as shown for the titration of phenylphosphonic acid **2** vs. KOH in Figure 2:

The deprotonation of each of the two P-OH functions led to a strong low field shift for the *ipso*-C1 carbon. For the remaining carbons high field shifts are observed, an effect decreasing in the order *para*-C4 > *meta*-C3/5 > *ortho*-C2/5.

Semi-empirical calculations with VAMP 4.4 using parameter set AM1 showed that the electron density at C1 increases with deprotonation in the order PhPO_3_H_2_ < PhPO_3_H^−^ < Ph-PO_3_^2−^, while the electron density of C4 decreases in this order [44,47]. In addition, the deprotonation of P-OH led to a decrease for all ^n^*J*_PC_ (n = 1 to 4). Particularly indicative is ^1^*J*_PC_ from the *ipso*-carbon C1, which reaches a minimum at total deprotonation. Numerical results for compound 2 are listed in Table 4:

Laborious and time-consuming single sample ^31^P{^1^H}- and ^13^C{^1^H}-NMR studies on phenylphosphonic acid **2** were performed, where δ_P_, δ_C_, and ^n^*J*_PC_ data are consistent with findings derived from the automated titrations presented in this paper [17].

### 2.2. Comparison of Aliphatic and Aromatic Aminophosphonic Acids

**Scheme 3:**
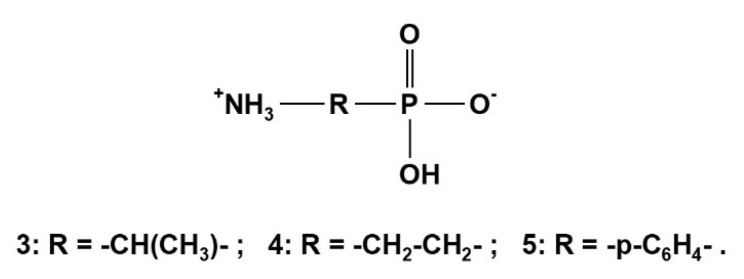
Aminophosphonic acids **3** to **5**.

α-Aminoethylphosphonic acid (α-Ala-P) **3** [29,44], β-aminoethylphosphonic acid (β-Ala-P, CILIATIN) **4** [29], and *p*-aminophenylphosphonic acid **5** [29,30,43] shown in Scheme 3 will be studied as examples in the following section.

#### 2.2.1. Aliphatic Aminophosphonic Acids

Aminophosphonic acids NH_2_-R-PO_3_H_2_, such as examples **3** to **5**, exist as betainic forms ^+^NH_3_-R-PO_3_H^−^ in solid and in solution state. Protolytic equilibria of aminophosphonic acids are described by macroscopic and microscopic formalisms as shown in Table 5 below:

If R is aliphatic (e.g., in **3** and **4**), the deprotonation takes place following route a): ^+^NH_3_-R-PO_3_H_2_ → ^+^NH_3_-R-PO_3_H^−^ → ^+^NH_3_-R-PO_3_^2−^ → NH_2_-R-PO_3_^2−^. But if R is aromatic (e.g., in **5**. R = *p*-C_6_H_4_-), the deprotonation dominantly will follow b): ^+^NH_3_-R-PO_3_H_2_ → ^+^NH_3_-R-PO_3_H^−^ → NH_2_-R-PO_3_H^−^ → NH_2_-R-PO_3_^2−^. Consistent conclusions are supported by a combination of potentiometric titrations and ^31^P{^1^H}-NMR-controlled titrations as shown for examples **3** to **5**, and in addition by ^13^C{^1^H}-NMR-controlled titrations for examples **3** and **4**. Owing to its low solubility, **5** was not suitable for ^13^C{^1^H}-NMR-controlled titrations.

Macroscopic dissociation constants pK_i_ of **3** and **4** are listed in Table 6. pK_i_ data of compounds **4** and **5** were discussed in [44,45,48,49,50].

Specific chemical shifts δ_P_ and gradients Δ for compounds **3** to **5** obtained by ^31^P{^1^H}-NMR-controlled titrations are given in Table 7:

The deprotonation of the P-OH groups led to high field shifts for δ_P_ connected with negative gradients. The final deprotonation of the NH_3_^+^ group gave rise to a low field shift for δ_P._ This effect is stronger in α-aminophosphonic acid **4** than in β-aminophosphonic acid **5**. Earlier results for chemical shifts δ_P_ of H_2_L, HL^−^ and L^2−^ species of **3** and **4** were mentioned in [5,45]. In addition, δ_P_ of H_3_L^+^ was accessible for **4** but not for **3**.

^13^C{^1^H}-NMR-controlled titrations of compounds **4** and **5** led to specific chemical shifts δ_C_, coupling constants ^1^*J*_PC_, and gradients Δ as listed in Table 8:

NMR was used to monitor the complex formation of aminophosphonic acids with biorelevant cations in homogeneous solutions. An instructive example is the ^31^P{^1^H}-NMR-controlled titration of CILIATIN/Mg^2+^ vs. NaOH where the formation of [MgL] and [MgHL]^+^ was monitored [44].

#### 2.2.2. Aromatic *p*-Aminophenylphosphonic Acid **5**

The deprotonation of PO_3_H^−^ in aliphatic aminophosphonic acids **3** and **4** is affiliated with a high field shift (gradients Δ_2_ are negative), while the deprotonation of the ammonium function ^+^NH_3_ leads to a low field shift (gradients Δ_3_ are positive).

**Scheme 4:**
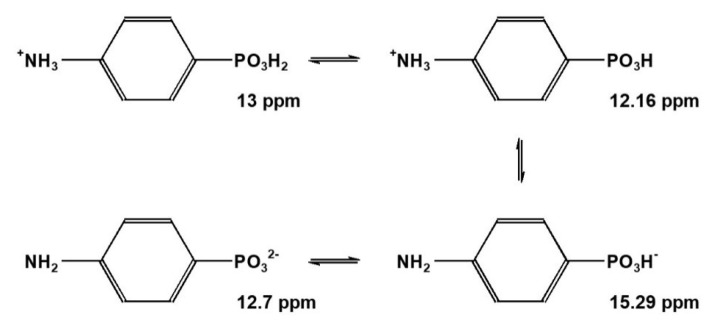
Specific chemical shifts δ_P_ [ppm] derived from the ^31^P{^1^H}-NMR-controlled retro titration of *p*-aminophenylphosphonic acid **5**. Δ_2_ = +3.13 ppm. Δ_3_ = −2.59 ppm.

The aromatic *p*-aminophenylphosphonic acid **5** exhibits a different pattern: while gradient Δ_2_ is positive, Δ_3_ is negative (Scheme 4).

But is it sufficient to assume a simple first-order macroscopic dissociation scheme for compound **5**? Deeper insight might be obtained from the microscopic dissociation scheme. In principle ^13^C{^1^H}-NMR-controlled titration should lead to specific chemical shifts and coupling constants ^n^*J*_PC_ indicative for microscopic dissociations species of **5**. But *p*-aminophenylphosphonic acid **5** is less soluble in water than the aliphatic aminophosphonic acids **3** and **4**. The S/N-ratio of ^13^C{^1^H}-NMR spectra of **5** is not sufficient to perform evaluable ^13^C{^1^H}-NMR-controlled titrations. In this situation, UV/VIS-controlled titration, which allows for lower concentrations suitable for conclusive measurements, will help to study both the macroscopic and the microscopic dissociation equilibrium of **5 [30]**. In addition, the parent compounds C_6_H_5_PO_3_H_2_ **2** and C_6_H_5_NH_2*_HCl **6** were compared. The following macroscopic pK_i_ data were found by potentiometric titration and listed in Table 9:

Those data point towards a dominating deprotonation sequence for **5** following ^+^NH_3_-R-PO_3_H_2_ → ^+^NH_3_-R-PO_3_H^−^ → NH_2_-R-PO_3_H^−^ → NH_2_-R-PO_3_^2−^. But is it justified to exclude the alternative route ^+^NH_3_-R-PO_3_H_2_ → ^+^NH_3_-R-PO_3_H^−^ → ^+^NH_3_-R-PO_3_^2−^ → NH_2_-R-PO_3_^2−^? Evaluating the macroscopic dissociation constants of **5** shows that between pH = 1.5 and pH = 10, only three macroscopic species exist: H_2_L, HL^−^, and L^2−^. The UV/VIS-controlled titration of **5** [30] showed that the maximum concentration for macroscopic HL^−^ is reached at pH = 5.75, consisting of two microdissociation species NH_2_-R-PO_3_H^−^ and ^+^NH_3_-R-PO_3_^2−^ in a ratio of 9:1. Thus, the results from the UV/VIS-controlled titration of **5** [30] confirm the dominance of NH_2_-R-PO_3_H^−^ as previously assumed for the macroscopic deprotonation sequence derived from the ^31^P{^1^H}-NMR-controlled titration of **5** [44].

### 2.3. Phosphonocarboxylic Acids HOOC-(CH_2_)_n_-PO_3_H_2_ **7a** to **7d** (n = 0 to 3)

**Scheme 5:**
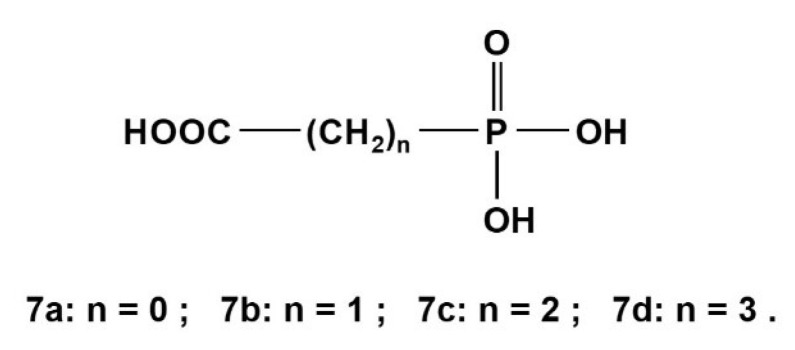
Phosphonocarboxylic acids **7a** to **7d**.

Phosphonocarboxylic acids HOOC-(CH_2_)_n_-PO_3_H_2_ gave rise to potentiometrically [4,44] and NMR-controlled titrations [4,28,31,44]. Those neutral acids of type H_3_L deprotonate dominantly in a sequence: HOOC-(CH_2_)_n_-PO_3_H_2_ → ^−^OOC-(CH_2_)_n_-PO_3_H^−^ → ^−^OOC-(CH_2_)_n_-PO_3_^2−^. Corresponding dissociations constants for compounds shown in Scheme 5 are listed in Table 10 below:

^13^C{^1^H}-NMR-controlled titrations yielded the specific chemical shift δ_C_ and coupling constants ^n^*J*_PC_ (n = 1 to 3) of phosphonocarboxylic acids HOOC-(CH_2_)_n_-PO_3_H_2_ (n = 0 to 3) **7a** to **7d** as listed in Table 11a. Gradients are given in Table 11b. Note: The deprotonation of P-OH groups and of C-OH led to a low field shift for all carbon atoms. Some chemical shifts and coupling constants ^1^*J*_PC_ of **7a** and **7c** were obtained and discussed in an early key paper [4].

#### 2.3.1. Compound **7c**: ^13^C{^1^H}-NMR-Controlled Titration of 3-Phosphonopropionic Acid HOOC-CH_2_-CH_2_-PO_3_H_2_ 7c.

**Figure 3:**
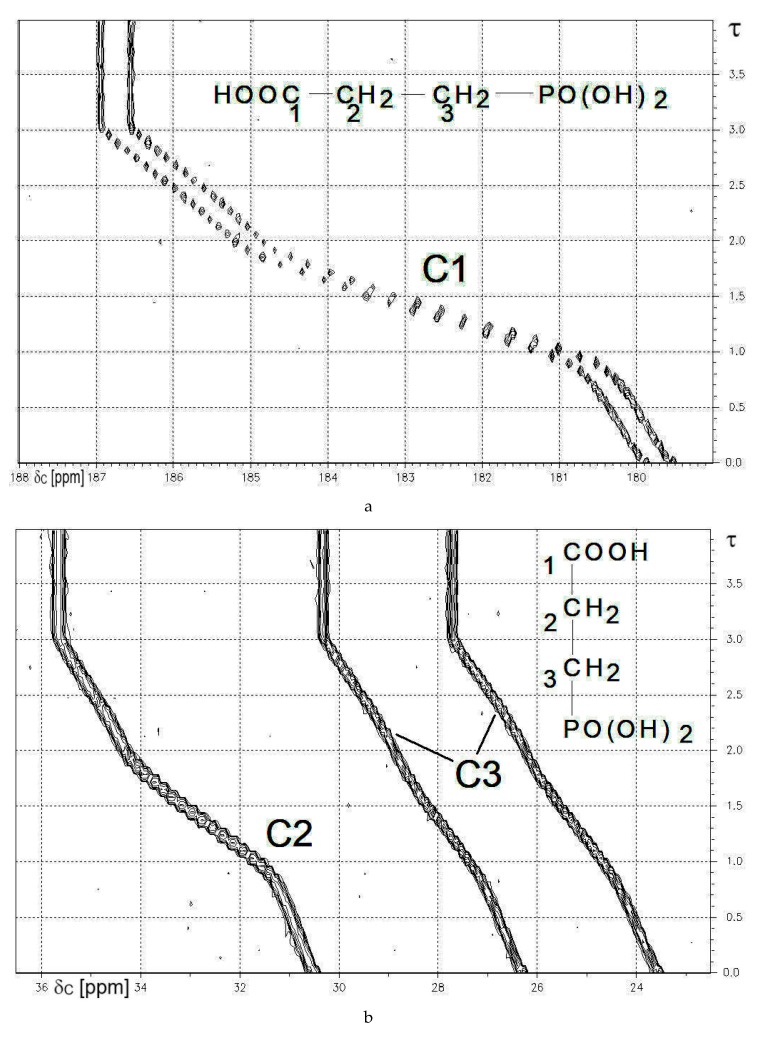

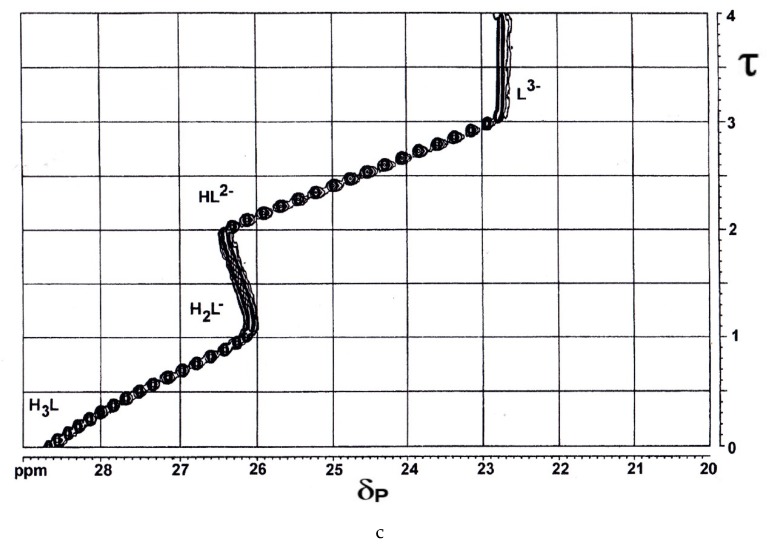
(**a**) ^13^C{^1^H}-NMR-controlled titration of 3-phosphonopropionic acid **7c** vs. NaOH. Contour plot showing the range of the carboxylic carbon C1. For experimental data, see Table 10. *X*-axis: δ_C_ [ppm]. *Y*-axis: degree of titration τ; (**b**) ^13^C{^1^H}-NMR-controlled titration of 3-phosphonopropionic acid **7c** vs. NaOH. Contour plot showing the range of the aliphatic carbons C2 and C3. For experimental data, see Table 10. *X*-axis: δ_C_ [ppm]. *Y*-axis: degree of titration τ; (**c**) 81 MHz ^31^P{^1^H}-NMR-controlled titration of 3-phosphonopropionic acid **7c** vs. NaOH *X*-axis: δ_P_ [ppm]. *Y*-axis: degree of titration τ. C_Titrand_: 0.010 mol/L of **7c**. C_Titrator_: 0.10 mol/L TMAOH. Digital resolution: 0.6 Hz/point in *x*-axis. [51].

3-Phosphonopropionic acid **7c** was chosen as an example to show practical results from ^13^C{^1^H}-NMR-controlled titrations (see Figure 3a,b below). The deprotonation of C-OH and of both P-OH functions induces low field shifts for C1, C2, and C3. Hence, the corresponding gradients are negative. Lorentzian deconvolution of ^13^C{^1^H} signals yielded ^n^*J*_PC_, where absolute values follow the sequence: ^1^*J*_PC_ >> ^3^*J*_PC_ > ^2^*J*_PC._ See Table 11a,b above.

The 81 MHz ^31^P{^1^H}-NMR-controlled titration of 3-phosphonopropionic acid **7c** vs. NaOH yielded Figure 3c. The deprotonation sequence reported in [4] corresponds to: HOOC-CH_2_-CH_2_-PO_3_H_2_ → HOOC-CH_2_-CH_2_-PO_3_H^−^ → ^−^OOC-CH_2_-CH_2_-PO_3_H^−^ → ^−^OOC-CH_2_-CH_2_-PO_3_^2−^. Deprotonation at PO_3_H_2_ or PO_3_H^−^ is affiliated with high field shifts of δ_P_, while deprotonation at HOOC induces a low field shift for δ_P_.

Specific chemical shifts δ_P_ for **7c** and corresponding anions together with gradients are listed in Table 12.

#### 2.3.2. ^19.^ F-NMR-Controlled Retro Titrations of Lithium Salts LiOOC-CH_2-n_F_n_-PO_3_Li_2_ **8a** and **8b**

**Scheme 6:**
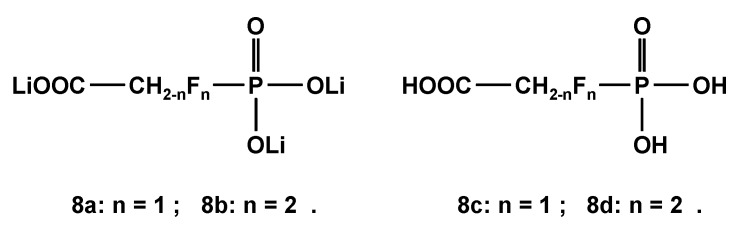
Trilithium salts LiOOC-CH_2-n_F_n_-PO_3_Li_2_ **8a** and **8b** and free acids HOOC-CH_2-n_F_n_-PO_3_H_2_ **8c** and **8d**.

The trilithium salts LiOOC-CH_2-n_F_n_-PO_3_Li_2_ (**8a** and **8b**; n = 1 and 2, Scheme 6) were used for retro titrations vs. HNO_3_, since the parent mono- and difluorophosphonoacetic acids **8c** and **8d** were not available for ^19^F-NMR- and ^31^P{^1^H}-NMR titrations. Corresponding dissociation constants pK_i_ of **8c** and **8d** were calculated as listed in Table 13, while chemical shifts δ_F_ and δ_P_ and coupling constants ^2^*J*_PF_ are given in Table 14. As expected, the introduction of fluorine into the skeleton of the parent phosphonoacetic acid led to lower pK_1_ and pK_2_ values. The deprotonation of P-OH groups induces a low field shift for δ_F_ in fluorinated phosphonic acids **8c** and **8d**.

#### 2.3.3. 2,4-Diphosphonobutane-1,2-Dicarboxylic Acid (DPBDC) **9**

Strong practical interests focused on polyfunctional phosphonocarboxylic acids, e.g., phosphonosuccinic acid (PBS), 1-phosphonopropane-1,2,3-tricarboxylic acid (PPTC), and 2-phosphonobutane-1,2,4-tricarboxylic acid (PBTC), which gave rise to analytical and NMR studies of protolytic and complex formation equilibria [42,43,44,53].

The following section will deal with 2,4-diphosphonobutane-1,2-dicarboxylic acid (DPBDC) **9** to demonstrate the potential of automated NMR titrations. Dissociation constants of this 6-valent acid DPBDC **9** were obtained from precision potentiometric [53] and by ^13^C{^1^H}-NMR-controlled titrations vs. NaOH [44].

**Scheme 7:**
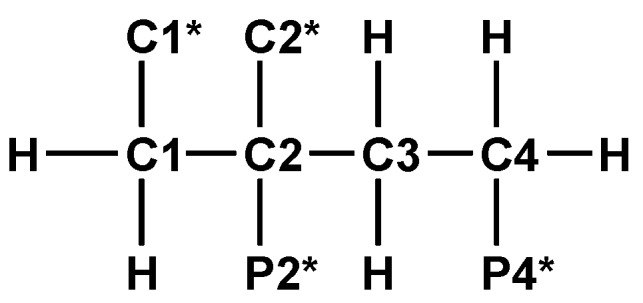
Spin enumeration in DPBDC 9 used for ^13^C{^1^H}-NMR C1* and C2* = COOH, COO^−^. P2* and P4* = PO_3_H_2_, PO_3_H^−^, PO_3_^2−^.

**Figure 4:**
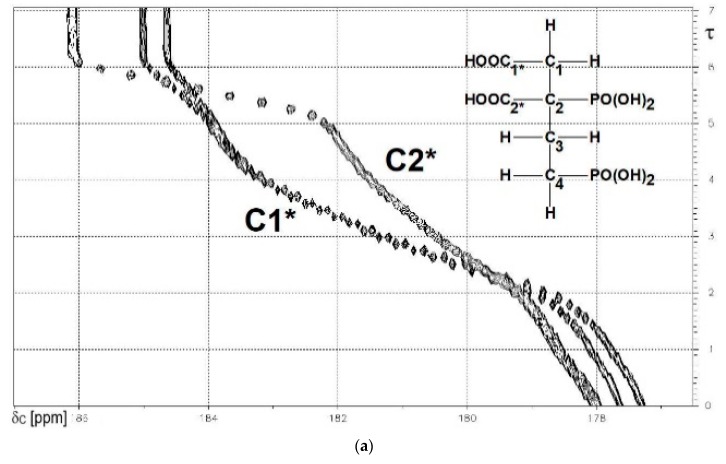

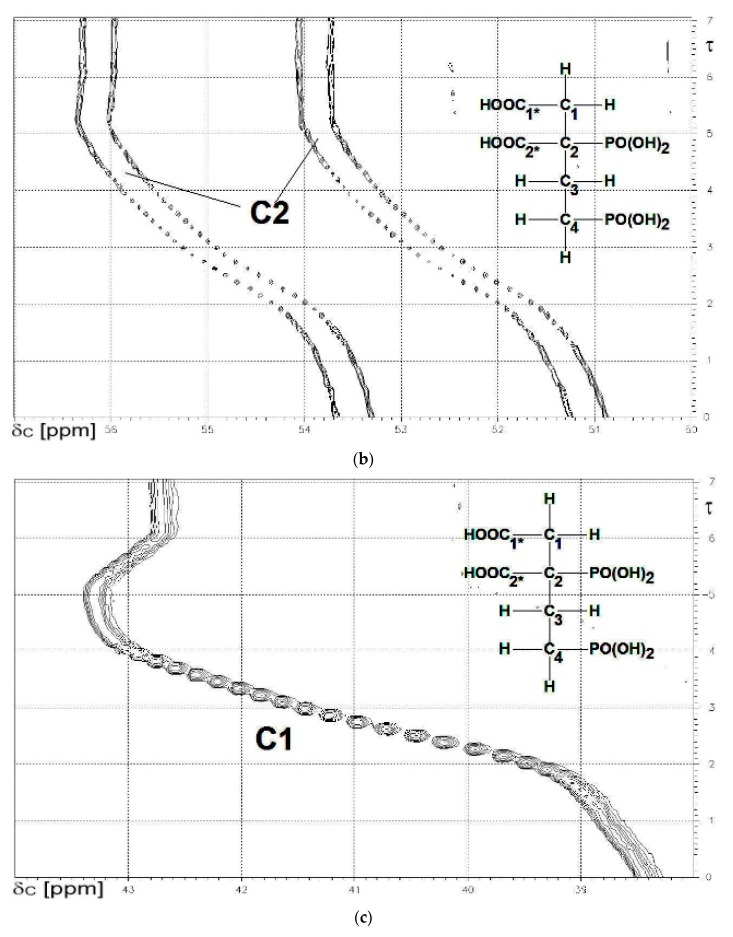

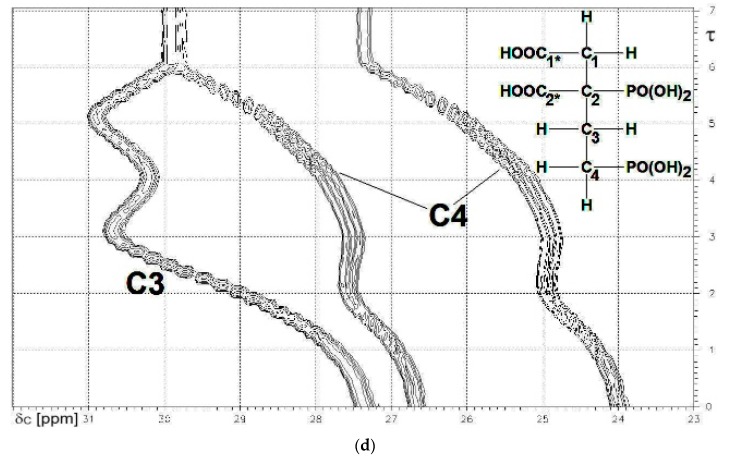
(**a**) The carboxylate functions C1* (note: ^3^*J*_PC_) and C2*. δ_C_ = 186 to 177 ppm; (**b**) The skeleton carbon C2 (note: ^1^*J*_PC_ and ^3^*J*_PC_). δ_C_ = 56 to 50 ppm.; (**c**) The skeleton carbon C1. δ_C_ = 44 to 38 ppm; (**d**) The skeleton carbons C3 and C4 (note: ^1^*J*_PC_). δ_C_ = 32 to 23 ppm.

^13^C{^1^H}-technique yielded Figure 4a–d. The spin enumeration used in subsequent tables and figures is given in Scheme 7:

Results for those six carbon atoms C1*, C2*, and C1 to C4 will be presented as (δ,τ))-contour plots in four separate spectral ranges shown in Figure 4a–d. Numerical results including specific chemical shifts δ_C_ and coupling constants ^n^*J*_PC_ of DPBDC are listed in Table 15 and Table 16.

##### Some Comments on DPBDC 9

**Scheme 8:**
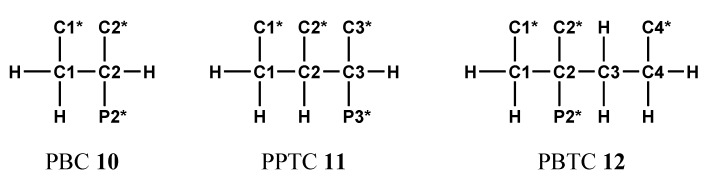
Phosphonopolycarboxylic acids **10** to **12** used for comparative ^13^C{^1^H}-NMR-controlled titrations.

A complicated example for NMR-controlled titration which needs some discussion is 2,4-diphosphonobutane-1,2-dicarboxylic acid DPBDC **9**. Measurements and data evaluation were performed according to the state of technique. But it is not possible to explain all the parameters found for compound **9** by comparison with data from analogous structural elements of HOOC-(CH_2_)n-PO_3_H_2_ (n = 1 to3) **7b** to **7d**, H_2_O_3_P-(CH_2_)_3_-PO_3_H_2_, and phosphonopolycarboxylic acids **10** to **12** shown in Scheme 8:

In a starting phase, 1D and 2D NMR techniques involving ^1^H-, ^1^H{^31^P}-, ^13^C-, ^13^C{^1^H}-, and C,H-COSY spectra were combined to assign the carbons C1*, C2*, C1 to C4 and phosphonate functions P2* and P4*.

For ^13^C{^1^H}-NMR-controlled titration, the deprotonation steps may be divided into three sections (see Table 15 and Figure 4a,d). For τ = 0 to 2 deprotonation PO_3_H_2_ → PO_3_H^−^ takes place, first at P2* and then at P4*. In the second section for τ = 2 to 4, the carboxylic units C1* and C2* are deprotonated. Finally for τ = 4 to 6 the deprotonation PO_3_H^−^ → PO_3_^2−^ takes place at P2* and P4*.

(1) Comments on Chemical Shifts δ_C_ of Carbon Atoms in DPBDC **9**

The deprotonation of PO_3_H_2_, PO_3_H^−^ and COOH functions in DPBDC **9** leads to a monotonous down field shift for δ_C_ C1* and C2* (see Figure 4a), while carbons C1 to C4 exhibit specific non-monotonous trends (see Figure 4b,d).

Since gradient Δ_6_ for δ_C_ (C1*) > Δ_5_ for δ_C_ (C1*), the final sixth deprotonation steps is affiliated with P2*. This conclusion is confirmed by Δ_6_ for δ_C_ (C2*) >> Δ_5_ for δ_C_ (C1*). Hence, the fifth deprotonation step of **9** is due to PO_3_H^−^ → PO_3_^2−^ of P4*. Dynamic chemicals shifts δ_C_ of C1* span a range from 177.5 to 184.61 ppm, while δ_C_ of C2* is found from 178 to 185.1, as shown in Figure 4a.

A tentative explanation may be found using Δ_3_ for δ_C_ (C1*) > Δ_4_ for δ_C_ (C1*) and Δ_3_ for δ_C_ (C2*) < Δ_4_ for δ_C_ (C1*). These findings imply that the carboxylic function C1* is more acidic than C*2.

Similar arguments for the relative acidity of C1* and C2* may be derived from the chemical shift δ_C_ of the skeleton carbon C2 (see Figure 4b). δ_C_ (C2) of H_6_L corresponds to 52.2 ppm, while the totally deprotonated form L^6−^ is found at 55 ppm. Deprotonation at C1* and C2* is characterized again by Δ_3_ of δ_C_ (C2) > Δ_4_ of δ_C_ (C2).

Chemical shifts δ_C_ of C3 span a range of 38.4 to 43.3 ppm. Surprisingly, the final deprotonation HL^5−^ → L^6−^, due to PO_3_H^−^ → PO_3_^2−^ of P2* reduces δ_C_ (C3) from 43.26 to 42.70 ppm. This is the first observation (within this context) of a negative gradient (Δ_6_ = −0.56 Hz) connected to deprotonation at a PO_3_H^−^ unit.

The situation is even more complex for the chemical shift δ_C_ of C2 covering a range from 27.4 to 30.9 ppm (Figure 4d). Two negative gradients are observed: Δ_4_ = −0.97 ppm for H_3_L^3−^ → H_2_L^4−^ due to deprotonation at C2* and Δ_6_ = −1.12 ppm for HL^5−^ → L^6−^ induced by deprotonation at P2*. For simpler compounds CH_3_-(CH_2_)_n_-COOH (n = 0 to 3) and HOOC-(CH_2_)_n_-COOH (n = 1 to 3), solely positive gradients were described [54].

In addition, we did not observe negative gradients for compounds **7b** to **7d** and **10** (PBC), but in **11** (PPTC) and in **12** (PBTC) [43].

The major RR/SS diastereomer of PPTC **11** exhibited a negative gradient Δ_5_ (C1) = −0.44 ppm for the final deprotonation step PO_3_H^−^ → PO_3_^2−^ at P3*. An upfield shift occurred, since δc (C1) of HL^4−^ = 42.17 ppm and δc (C1) of L^5−^ = 41.73 ppm. This effect might be due to opening of hydrogen bridges and conformational changes. In contrast here to is the minor RR/SS diastereomer of PPTC, it does not show a negative gradient Δ_5_ (C1) [42,44].

Weaker negative gradients Δ_5_ = −0.22 ppm are observed for chemical shifts δ_C_ of both carbons C1 and C3 in PBTC **12.** The final deprotonation PO_3_H^−^ → PO_3_^2−^ at P2* is affiliated with following data: δc (C1) of HL^4−^ = 43.54 ppm, δc (C1) of L^5−^ = 43.32 ppm, and δc (C3) of HL^4−^ = 32.60 ppm, δc (C3) of L^5−^ = 32.60 ppm. In contrast hereto carbons C2 and C4 in PBTC **12** exhibit positive gradients Δ_5_.

Those unexpected observations for chemical shifts δc in **9** and conformational aspects will be mentioned in the following section on coupling constants ^n^*J*_PC_ as well.

(2) Comments on Coupling Constants nJPC (n = 1 to 3) of DPBDC **9**

**Figure 5:**
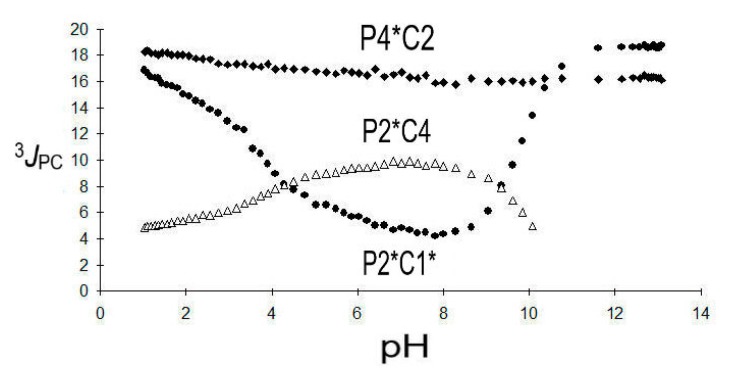
Three coupling constants ^3^*J*_PC_ (P2*C1*), ^3^*J*_PC_ (P2*C4), and ^3^*J*_PC_ (P4*C12) in DPBDC **9**.

**Scheme 9:**
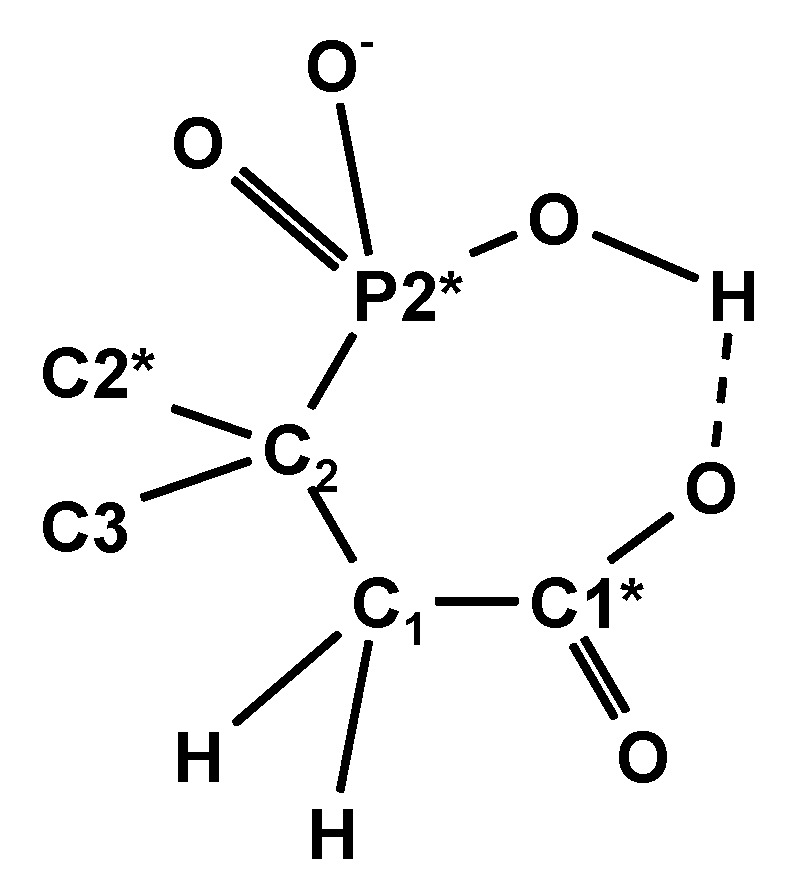
Tentative hydrogen bridges for protolytic species H_3_L^3−^ to HL^5−^.

The vicinal coupling ^3^*J*_PC_ (P2*C1*) is remarkably sensitive towards the protonation state (see Figure 5):

For the protolytic species H_6_L to H_2_L^4−^ of **9,** a decrease in ^3^*J*_PC_ (P2*C1*) from 17.5 Hz down to a minimum of 4.3 Hz is observed, followed by an increase from 5.3 Hz to 18.6 Hz due to HL^5−^ and finally L^6−^. Between pH = 7 and 8, a maximum of the protolytic species H_2_L^4−^ is expected, while HL^5−^ dominates around pH = 9. Those observations indicate changes of the dihedral angle of P2*-C2-C1-C1* possibly involving hydrogen bridges as indicated by Scheme 9 below:

A corresponding bridge -C1-P-O---H---O-P-C2- was discussed for the HL^3−^ species of ethane-1,2-bisphosphonic acid [44].

^2^*J*_PC_ (P2*C1), ^2^*J*_PC_ (P2*C3), ^2^*J*_PC_ (P2*C2*), and ^2^*J*_PC_ (P4*C3) were not resolved in ^13^C{^1^H}-NMR spectra obtained by NMR-controlled titrations.

^3^*J*_PC_ (P2*C4) shows a monotonous increase from 4.7 Hz to a maximum of 9.9 Hz for the sequence H_6_L to H_2_L^4−^ followed by a decrease to 9.4 Hz for HL^5−^. This observation points towards an increase in the dihedral angle in P2*-C2-C3-C4.

^3^*J*_PC_ (P4*C2) is less sensitive towards deprotonation but larger than ^3^*J*_PC_ (P2*C4) and found in a range from 18.4 to 16.3 Hz possibly indicating a tendency towards trans-conformation of the fragment P4*-C4-C3-C2. For comparison, ^3^*J*_PC_ in HOOC-(CH_2_)_3_-PO_3_H_2_ **7d** appeared in a corresponding range from 17.2 to 17.9 Hz.

**Figure 6:**
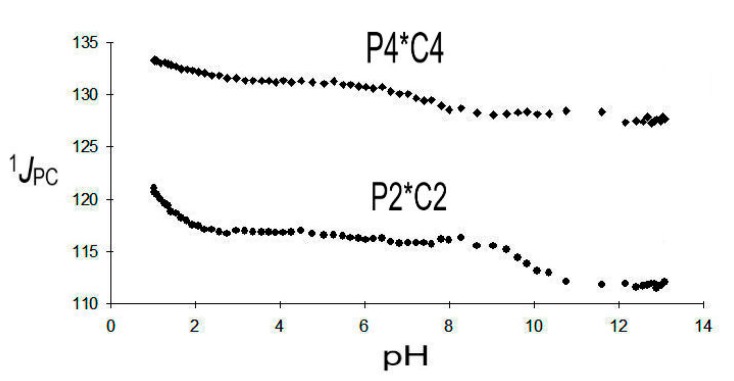
^1^*J*_PC_ (P2*C2) and ^1^*J*_PC_ (P4*C4) of DPBDC **9**.

^1^*J*_PC_ (P2*C2), ranging from 124.2 to 111.7 Hz, is markedly smaller than ^1^*J*_PC_ (P4*C4), which is observed from 134.0 to 127.7 Hz. A ^1^*J*_PC_, pH diagram is given in Figure 6 below:

^1^*J*_PC_ (P2*C2) is very indicative and selective for the deprotonation of the phosphonic functions PO_3_H_2_ → PO_3_H^−^ and PO_3_H^−^ → PO_3_^2−^. It indicates that the first deprotonation (pK_1_ = 1.07) of DPBDC **9** takes place at P2* with a strong gradient Δ_1_^1^*J*_PC_ (P2*C2) = −7.4 Hz. The final deprotonation (pK_6_ = 11.62) is affiliated with P2* as well as indicated by Δ_6_^1^*J*_PC_ (P2*C2) = −4.1 Hz. This assignment leaves pK_2_ = 2.73 and pK_5_ = 8.95 to the deprotonation of P4*. Deprotonation at the carboxylic groups (pK_3_ = 4.62) and pK_4_ = 7.05) does not significantly affect ^1^*J*_PC_ (P2*C2) and ^1^*J*_PC_ (P4*C4).

Results on ^1^*J*_PC_ (P2*C2) of **9** are consistent with observations on phosphonosuccinic acid **10**, where ^1^*J*_PC_ (P2*C2) is found in a range from 133.7 to 112.1 Hz. Strong gradients Δ_1_ = −17.1 Hz and Δ_4_ = −4.5 Hz are affiliated with the deprotonation of P2*, while deprotonations of C1* and C2* do not significantly influence ^1^*J*_PC_ (P2*C2).

## 3. Conclusions

Automated NMR-controlled titrations efficiently combine ^1^H, ^13^C-, ^19^F-, and ^31^P-NMR spectroscopy with potentiometric titrations to determine dissociation constants, specific chemicals shifts and coupling constants. Results are presented in two-dimensional plots, where NMR parameters (chemical shifts, coupling constants) are correlated with analytical parameters (pH, degree of titration τ). High digital resolution and high S/N are achieved in time- and material-saving measurements. These hyphenated techniques are powerful instruments to identify the structure and purity of research and industrial compounds. Limitations of accuracy due to the nature of glass electrodes occur at very low and very high pH values, obscuring the lower and higher pK_i_ values. In those situations, single sample NMR methods are recommended. ^13^C{^1^H}-NMR-controlled titrations may be used for conformational analysis. For more complicated structures, additional studies using pH-dependent high-resolution ^1^H- and ^1^H{^31^P}-NMR spectra, X-ray diffraction of selected salts and molecular modelling of acids and anions are needed to solve details of conformational problems. The latter topics are laborious and beyond the scope of this paper.

## 4. Experimental

Details of the hardware and software used in NMR-controlled titration together with references for this field are described in [31,32]. Most model compounds were obtained from external sources listed under the acknowledgements.

## Figures and Tables

**Table 1:** Some examples for NMR-controlled titrations of phosphonic acids, phosphinic acids, and carboxylic acids as discussed in [32]. ^1)^ Retro titration; ^2)^ Micro dissociation; ^3)^ Second-order ^1^H-NMR spin systems.

Examples	NMR	Remarks
**Phosphonic acids**		
CH_3_P(O)(OH)_2_	^31^P{^1^H}	
LiOOC-CH_2_-P(O)(OLi)_2_	^31^P{^1^H}	^1)^
(HO)_2_(O)P-CH_2_-CH_2_-P(O)(OH)_2_	^13^C{^1^H}	
CH_3_-C(OH)[P(O)(OH)_2_]_2_, HEDP, etidronic acid	^31^P{^1^H}	
NH_2_-CH_2_-CH_2_-C(OH)(P(O)(OH)_2_)_2_, pamidronic acid	^31^P{^1^H}	
HOOC-CH_2_-CH(COOH)-CH(COOH)-P(O)(OH)_2_, PPTC	^31^P{^1^H}	
**Phosphinic acids**		
(CH_3_)_2_P(O)OH	^31^P{^1^H}	
HOOC-CH_2_-CH_2_-P(CH_3_)(O)OH	^13^C{^1^H}, ^1^H	^2)^
HO(O)(CH_3_)P-CH_2_-CH_2_-P(CH_3_)(O)OH	^13^C{^1^H}	
HO(O)(CH_3_)P-CH_2_-CH_2_-C(H)(NH_2_)COOH	^1^H	^2, 3)^
**Carboxylic acids**		
CH_3_COOH	^13^C{^1^H}	
CH(CH_3_)_2_-CH_2_-CH(NH_2_)-C(O)-NH-CH(CH_3_)-COOH, peptide Leu-Ala	^13^C{^1^H}	
CH_2_=CF-CH_2_-C(CH_3_)(NH_2_)-COOH	^19^F	

**Table 2:** Specific chemical shifts δ_C_, δ_P_ and coupling constants ^1^*J*_PC_ and ^2^*J*_PH_ of methanephosphonic acid **1** were obtained by ^13^C{^1^H}-, ^31^P{^1^H}-, and ^31^P-NMR-controlled titrations in H_2_O. Δ_i_ = δ(H_n-i_L) − δ(H_n+1-i_L) or Δ_i_ = ^n^*J*_PC_(H_n-i_L) − ^n^*J*_PC_(H_n+1-i_L), respectively. i = 1 to n. n = 2. Experimental data: C_Titrand_: a) 0.269 mol/L. b) 0.01220 mol. c) 0.0095 mol/L. C_Titrator_: a) 4.82 mol/L KOH. b) 0.0971 mol/L NaOH. c) 0.0971 mol/L NaOH. d) Early data from results from titration vs. KOH [45].

	1 in H_2_O					
Method	^13^C{^1^H}		^31^P{^1^H}	^31^P		^31^P{^1^H}
**Exp.**	a)		b)	c)		d)
**Species**	**δ_C_**	**^1^*J*_PC_**	**δ_P_**	**δ_P_**	**^2^*J*_PH_**	**δ_P_**
	[ppm]	[Hz]	[ppm]	[ppm]	[Hz]	[ppm]
**H_2_L**	14.27	135.92	33.03	33.03	−17.65	31.76
**HL^−^**	15.53	133.82	24.79	24.79	−16.52	24.94
**L^2−^**	16.61	129.95	21.08	21.08	−15.52	20.94
**Gradients**	**δ_C_**	**^1^*J*_PC_**	**δ_P_**	**δ_P_**	**^2^*J*_PH_**	**δ_P_**
Δ**_1_**	1.26	−2.10	−8.24	−8.24	1.13	
Δ**_2_**	1.08	−3.87	−3.71	−3.71	1.00	
**pK_i_**						
**pK_1_**	2.27		2.06	2.00		2.33
**pK_2_**	7.85		7.66	7.68		7.78

**Table 3:** Specific chemical shifts δ_P_ [ppm], gradients [ppm] and dissociation constants pK_i_ from ^31^P{^1^H}-NMR-controlled titration of phenylphosphonic acid **2** vs. TMAOH and NaOH. Exp.: C_Titrator_: a) 0.09894 mol/L TMAOH. b) 0.09925 mol/L NaOH. C_Titrand_: a) 0.02 mol/L **3**. b) 0.008 mol/L **2**. Shifts and gradients given in ppm.

	Phenylphosphonic Acid 2	
	vs. TMAOH	vs. NaOH
	a)	b)
**δ_P_(H_2_L)**	18.39	17.77
**δ_P_(HL^−^)**	13.77	13.75
**δ_P_(L^2−^)**	11.69	11.72
Δ**_1_**	−4.62	−4.02
Δ**_2_**	−2.08	−2.03
**pK_1_**	1.74	1.86
**pK_2_**	7.28	7.16

**Table 4:** Specific chemical shifts δ_C_ [ppm] and coupling constants ^n^*J*_PC_ (n = 1 to 4) [Hz] for the ^13^C{^1^H}-NMR-controlled titration of phenylphosphonic acid **2** vs. KOH. pK_1_ = 1.75. pK_2_ = 6.92. Experimental data: C_Titrand_: a) 0.237 mol/L **2**. C_Titrator_: 4.53 mol/L KOH.

	δ_C_ and ^n^*J*_PC_ for Species			Gradients	
Parameters	H_2_L	HL^−^	L^2−^	Δ_1_	Δ_2_
**δ_C_ C1**	133.66	138.15	143.83	+4.48	+5.69
**δ_C_ C2/6**	133.68	133.45	133.43	−0.24	−0.02
**δ_C_ C3/5**	131.99	131.59	130.95	−0.40	−0.64
**δ_C_ C4**	135.66	134.03	131.98	−1.58	−2.06
**^1^*J*_PC_**	183.48	177.02	167.32	−6.46	−9.70
**^2^*J*_PC_**	10.51	9.72	8.79	−0.79	−0.93
**^3^*J*_PC_**	14.84	13.90	12.65	−0.94	−1.25
**^4^*J*_PC_**	3.08	2.91	2.74	−0.17	−0.17

**Table 5:** Macroscopic and microscopic dissociation species of aminophosphonic acids.

Dissociation Species		
Macroscopic	Microscopic	
**H_3_L^+^**	^+^NH_3_-R-PO_3_H_2_	
**H_2_L**	^+^NH_3_-R-PO_3_H^−^	NH_2_-R-PO_3_H_2_
**HL^−^**	^+^NH_3_-R-PO_3_^2−^	NH_2_-R-PO_3_H^−^
**L^2−^**		NH_2_-R-PO_3_^2−^

**Table 6:** Macroscopic dissociation constants pK_i_ of compounds α-Ala-P **3** and β-Ala-P **4** obtained by ^13^C{^1^H}- and ^31^P{^1^H}-NMR-controlled titrations and by potentiometric titrations [4a]. Note: pK_3_ − pK_2_ > 3 and pK_2_ − pK_1_ > 3 for compounds **3** and **4**. Exp.: **3:** C_Titrand_: a) 0.0867 mol/L **3** + 0.0834 mol/L HNO_3_. b) and c) 0.005 mol/L **3** + 0.00476 HNO_3_ + 0.0917 mol/L NaNO_3_. C_Titrator_: a) 3.98 mol/L NaOH. b) and c): 0.100 mol/L NaOH. **4**: C_Titrand_: d) 0.139 mol/L **4** + 0.139 mol/L HNO_3_. e) 0.010 mol/L **4** + 0.010 mol/L HNO_3_. f) 0.010 mol/L **4** + 9.747 mmol/L HNO_3_. g) 0.005 mol/L **4** + 0.005 mol/L HNO_3_ + 0.100 mol/L TMACl. C_Titrator_: d) 0.98 mol/L NaOH. e): 0.0991 mol/L TMAOH. f): 0.0993 mol/L NaOH. g): 0.099 mol/L TMAOH.

	3			4			
	^13^C{^1^H} a	^31^P{^1^H} b	Pot. c	^13^C{^1^H} d	^31^P{^1^H} e	^31^P{^1^H} f	Pot. g
	NaOH	NaOH	NaOH	NaOH	TMAOH	NaOH	TMAOH
**pK_1_**	0.70	0.31	0.3	1.02	1.22	1.26	1.14
**pK_2_**	5.72	5.63	5.58	6.38	6.23	6.24	6.34
**pK_3_**	10.64	10.21	10.28	11.50	11.06	11.08	11.06

**Table 7:** Specific chemical shift δ_P_ [ppm] and gradients Δ [ppm] for compounds **3** to **5** [44]. *) Not iterated. Experimental details for **3** and **4** are given in Table 6. Titrator: a) NaOH; b) TMAOH; c) for **5** were used: C_Titrand_ = 1.6953 mol/L **5** and 3.6935 mol/L TMAOH. Titrator = 0.09993 mol/L HCl.

	3	4		5
	^31^P{^1^H} a	^31^P{^1^H} b	^31^P{^1^H} a	^31^P{^1^H} c
**Species**	**δ_P_**	**δ_P_**	**δ_P_**	**δ_P_**
**H_3_L^+^**	15 *	22.9	23.4	13 *
**H_2_L**	14.92	19.29	19.36	12.16
**HL^−^**	13.08	16.80	16.81	15.29
**L^2−^**	22.25	19.39	19.72	12.70
**Gradients**				
Δ**_1_**	−0.08	−3.61	−4.04	−0.84
Δ**_2_**	−1.84	−2.49	−2.55	+3.13
Δ**_3_**	+9.17	+2.59	+3.91	−2.59

**Table 8:** Specific chemical shifts δ_C_ [ppm], coupling constants ^1^*J*_PC_ [Hz], and corresponding gradients Δ for α-aminoethanephosphonic acid (α-Ala-P) **3** and β-aminoethanephosphonic acid (β-Ala-P, CILIATIN) **4**. Spin enumerations: **3**: C2-C1(N)-P; **4**: (N)C2-C1-P. ^1^*J*_PC_ shows a minimum for species HL^−^ of **3** and **4**. ^2^*J*_PC_ was not resolved for compounds **3** and **4**.

	3 in H_2_O			4 in H_2_O		
**Species**	**δ_C_(C1)**	**^1^*J*_PC_**	**δ_C_(C2)**	**δ_C_(C1)**	**^1^*J*_PC_**	**δ_C_(C2)**
**H_3_L^+^**	46.80	151.5	16.0	27.50	137.4	37.09
**H_2_L**	47.70	143.8	16.43	28.73	131.4	38.22
**HL^−^**	49.07	134.5	17.23	29.47	124.8	39.28
**L^2−^**	48.15	138.0	19.79	35.45	126.5	39.76
**Gradients**						
Δ**_1_**	+0.9	−8.7	+0.43	+1.23	−6.0	+1.13
Δ**_2_**	+1.37	+9.3	+0.80	+0.74	−6.6	+1.04
Δ**_3_**	−0.92	+3.5	+2.55	+5.98	+1.3	+0.47

**Table 9:** Dissociation constants of compounds *p*-aminophenylphosphonic acid **5**, phenylphosphonic acid **2**, and anilinium hydrochloride **6.**

	5	2	6
**pK_1_**	0.44	1.88	4.68
**pK_2_**	3.95	7.15	
**pK_3_**	7.56		

**Table 10:** Dissociation constants of phosphonocarboxylic acids HOOC-(CH_2_)_n_-PO_3_H_2_ (n = 0 to 3) **7a** to **7d** [8,44]. Experimental data: ^a)^ C_Titrand_: 0.153 mol/L FOSCARNET (trisodium phosphonoformate hexahydrate), C_Titrator_: 2.002 mol/L HNO_3_, retro titration; ^b)^ C_Titrand_: 0.220 mol/L **7b**, C_Titrator_: 0.980 mol/L NaOH. ^c)^ C_Titrand_: 0.200 mol/L **7c**, C_Titrator_: 3.986 mol/L NaOH. ^d)^ C_Titrand_: 0160 mol/L **7d**, C_Titrator_: 3.986 mol/L NaOH.

	HOOC-(CH_2_)_n_-PO_3_H_2_					
	7a	7a [4]	7b	7c	7c [4]	7d
	n = 0 ^a^	n = 0	n = 1 ^b^	n = 2 ^b^	n = 2	n = 3 ^b^
**pK_1_**	0.78	1.7 ± 0.1	1.22 ± 0.166	2.58 ± 0.013	2.26 ± 0.04	2.276 ± 0.006
**pK_2_**	3.60	3.59 ± 0.02	4.942 ± 0.004	4.633 ± 0.004	4.63 ± 0.02	4.776 ± 0.004
**pK_3_**	7.57	7.56 ± 0.02	8.099 ± 0.003	7.738 ± 0.003	7.75 ± 0.02	7.969 ± 0.003

**Table 11:** (**a**). Specific chemical shifts δ_C_ [ppm] and coupling constants ^n^*J*_PC_ [Hz] of phosphonocarboxylic acids HOOC-(CH_2_)_n_-PO_3_H_2_ (n = 0 to 3) **7a** to **7d**. For experimental data, see preceding Table 10. Remarks: n. r. = not resolved; (**b**). Gradients Δ_i_ of specific chemical shifts δ_C_ [ppm] and coupling constants ^n^*J*_PC_ [Hz] of phosphonocarboxylic acids HOOC-(CH_2_)_n_-PO_3_H_2_ (n = 0 to 3) **7a** to **7d**. For experimental data, see preceding Table 10.

(**a**)										
**7**	**n**	**Species**	**δ_C_(C1)**	**δ_C_(C2)**	**δ_C_(C3)**	**δ_C_(C4)**	**^1^*J*_PC_**	**^2^*J*_PC_**	**^3^*J*_PC_**	**^4^*J*_PC_**
**a**	**0**	**H_3_L**	176.8				246.6			
		**H_2_L^−^**	178.7				236.7			
		**HL^2−^**	181.8				231.8			
		**L^3−^**	187.3				220.0			
**b**	**1**	**H_3_L**	172.91	37.68			128.6	n. r.		
		**H_2_L^−^**	175.44	39.30			117.8	n. r.		
		**HL^2−^**	178.85	41.64			119.2	n. r.		
		**L^3−^**	181.74	43.50			112.6	n. r.		
**c**	**2**	**H_3_L**	179.25	30.09	24.51		138.5	3.6	17.3	
		**H_2_L^−^**	180.68	31.40	25.91		135.1	3.2	17.8	
		**HL^2−^**	185.01	34.27	27.50		133.0	4.1	18.7	
		**L^3−^**	186.71	35.61	28.99		130.3	3.6	19.8	
**d**	**3**	**H_3_L**	180.38	36.67	20.44	28.19	135.2	4.0	17.4	n. r.
		**H_2_L^−^**	181.12	37.46	21.58	29.74	133.5	3.8	17.2	n. r.
		**HL^2−^**	185.86	41.45	23.13	30.46	132.5	3.9	17.7	n. r.
		**L^3−^**	186.41	42.01	24.08	31.92	130.1	3.4	17.9	n. r.
(**b**)										
**7**	**n**	**Gradients**	**δ_C_(C1)**	**δ_C_(C2)**	**δ_C_(C3)**	**δ_C_(C4)**	**^1^*J*_PC_**		**^2^*J*_PC_**	**^3^*J*_PC_**
**a**	**0**	Δ**_1_**	+1.9				−9.9			
		Δ**_2_**	+3.1				−4.9			
		Δ**_3_**	+5.5				−11.8			
**b**	**1**	Δ**_1_**	+2.53	+1.62			−10.8			
		Δ**_2_**	+3.41	+2.34			+1.4			
		Δ**_3_**	+2.89	+1.86			−6.6			
**c**	**2**	Δ**_1_**	+1.43	+1.31	+1.40		−3.4		−0.3	0.5
		Δ**_2_**	+4.33	+2.87	+1.59		−2.1		0.9	0.9
		Δ**_3_**	+1.70	+1.34	+1.49		−2.7		−0.5	1.1
**d**	**3**	Δ**_1_**	+0.74	+0.79	+1.14	1.56	−1.7		−0.2	−0.2
		Δ**_2_**	+4.74	+3.99	+1.55	0.72	−1.0		0.1	0.5
		Δ**_3_**	+0.55	+0.56	+0.95	1.46	−2.4		−0.5	0.2

**Table 12:** Gradients Δ_i_ of specific chemical shifts δ_P_ [ppm] of **7c**. Experimental data: C_Titrand_: 0.010 mol/L of **7c**. C_Titrator_: 0.10 mol/L TMAOH.

Shifts	δ_P_	Error
**H_3_L**	29.93	±0.25
**H_2_L^−^**	24.56	±0.02
**HL^2−^**	25.88	±0.01
**L^3−^**	22.06	±0.01
**Gradients**		
**Δ_1_**	−5.37	
**Δ_2_**	+1.32	
**Δ_3_**	−3.82	

**Table 13:** Dissociation constants of fluorinated phosphonocarboxylic acids HOOC-CH_2-**n**_F**_n_**-PO_3_H_2_ (**8c** and **8d**; n = 1 and 2) obtained by the retro titration of LiOOC-CH_2-**n**_F**_n_**-PO_3_Li_2_ (**8a** and **8b**; n = 1 and 2) vs. HClO_4_. Experimental data: C_Titrand_: 0.85 mol/L **8a** or **8b** resp. C_Titrator_: 0.3928 mol/L HClO_4_. No ion buffer.

	HOOC-CH_2-n_F_n_-PO_3_H_2_		
	8c n = 1	8d n = 2	
	[44]	[44]	[52]
**pK_1_**	1.05	0.52	1.30
**pK_2_**	3.43	2.22	1.95
**pK_3_**	7.08	6.36	6.16

**Table 14:** Chemical shifts δ_F_ [ppm], coupling constants ^2^*J*_PF_ [Hz] and corresponding gradients of fluorinated phosphonocarboxylic acids HOOC-CH_2-**n**_F**_n_**-PO_3_H_2_ (**8c** and **8d**, n = 1 and 2) obtained by ^19^F-NMR-controlled titrations vs. HNO_3_ of LiOOC-CH_2-**n**_F**_n_**-PO_3_Li_2_ (**8c** and **8d**, n = 1 and 2). For experimental data, see Table 13. δ_F_ is virtually referenced to δ_F_(CF^35^Cl_2_^37^Cl) = 0 ppm.

HOOC-CH_2-n_F_n_-PO_3_H_2_				
	8c	8d	8c	8d
	n = 1	n = 2	n = 1	n = 2
	^19^F		^19^F	
**Species**	**δ_F_**	**δ_F_**	**^2^*J*_PF_**	**^2^*J*_PF_**
**H_3_L**	−38.27	44.24	67.8	76.5
**H_2_L^−^**	−38.72	50.24	65.5	88.6
**HL^2−^**	−29.31	53.05	70.3	92.8
**L^3−^**	−27.15	55.24	63.4	82.0
**Gradients**				
**Δ_1_**	−0.45	+6.00	−2.3	+12.1
**Δ_2_**	−9.41	+2.81	+4.8	+4.2
**Δ_3_**	−8.16	+2.19	−6.9	−10.8

**Table 15:** Dissociation constants of 2,4-diphosphonobutane-1,2-dicarboxylic acid (DPBDC) as obtained from ^13^C{^1^H}-NMR-controlled and potentiometric titrations vs. NaOH. * Concentrations given in mol/L.

	^13^C{^1^H} NMR [44]	Potentiometric [44]	Potentiometric [53]
**pK_1_**	1.07	0.6	1.806 ± 0.066
**pK_2_**	2.73	2.42	2.250 ± 0.021
**pK_3_**	4.82	4.32	4.078 ± 0.005
**pK_4_**	7.05	6.46	6.562 ± 0.004
**pK_5_**	8.95	8.18	8.664 ± 0.006
**pK_6_**	11.62	10.75	12.839 ± 0.007
**C_Titrand_**	0.262 (DPBDC) *	0.0050 (DPBDC) *	0.01119 (DPBDC) *
**C_Titrator_**	3.986 (NaOH) *	0.0975 (NaOH) *	0.09863 (TMAOH) *
**C_Ion buffer_**	0	0.1 (NaCl) *	0.09863 (TMANO_3_) *

**Table 16:** Specific chemical shifts δ_C_ [ppm], coupling constants ^n^*J*_PC_ [Hz] and gradients Δ_i_ [ppm] of 2,4-diphosphonobutane-1,2-dicarboxylic acid DPBDC **9**. For experimental data, see Table 15.

	C1	C2			C3	C4			C1*		C2*
Species	δ_C_	δ_C_	^1^ *J*_PC_	^3^ *J*_PC_	δ_C_	δ_C_	^1^ *J*_PC_	^3^ *J*_PC_	δ_C_	^3^ *J*_PC_	δ_C_
**H_6_L**	38.29	52.19	124.2	18.4	27.26	25.11	134.0	4.7	177.23	17.5	177.53
**H_5_L^−^**	38.70	52.42	116.8	18.2	27.51	25.56	132.4	5.1	177.82	15.9	178.66
**H_4_L^2−^**	39.38	52.98	116.9	17.3	29.09	26.34	131.3	6.3	178.85	12.5	179.18
**H_3_L^3−^**	41.78	54.06	116.7	16.8	30.95	26.15	131.3	9.0	181.69	6.4	180.70
**H_2_L^4−^**	43.28	54.66	115.9	16.6	29.98	26.54	130.3	9.9	183.46	4.3	181.53
**HL^5−^**	43.26	55.04	115.8	15.9	30.99	27.51	128.3	9.4	184.08	5.3	182.53
**L^6−^**	42.70	55.01	111.7	16.3	29.87	28.65	127.7	-	184.82	18.9	186.15
**Gradients**											
**Δ_1_**	+0.41	+0.23	−7.4	−0.2	+0.25	+0.45	−1.6	+0.4	+0.59	−1.6	+1.13
**Δ_2_**	+0.68	+0.56	+0.1	−0.9	+1.58	+0.78	−1.1	+1.2	+1.03	−3.4	+0.52
**Δ_3_**	+1.40	+1.08	−0.2	−0.5	+1.86	−0.19	0.0	+2.7	+2.84	−6.1	+1.52
**Δ_4_**	+1.50	+0.60	−0.8	−0.2	−0.97	+0.39	−1.0	+0.9	+1.77	−2.1	+0.83
**Δ_5_**	−0.02	+0.38	−0.1	−0.7	+1.01	+0.97	−2.0	−0.5	+0.62	+1.0	+1.00
**Δ_6_**	−0.56	−0.03	−4.1	+0.4	−1.12	+1.14	−0.6	-	+0.74	+13.6	+3.62
